# Current and emerging treatment strategies for *Mycobacterium avium* complex pulmonary disease: a narrative review

**DOI:** 10.12771/emj.2025.00080

**Published:** 2025-03-26

**Authors:** Chiwook Chung

**Affiliations:** Division of Pulmonary, Allergy and Critical Care Medicine, Department of Internal Medicine, Hallym University Dongtan Sacred Heart Hospital, Hallym University College of Medicine, Hwaseong, Korea

**Keywords:** Bacterial drug resistance, Ethambutol, Lung diseases, *Mycobacterium avium* complex, Tuberculosis

## Abstract

The *Mycobacterium avium* complex (MAC), comprising *M. avium* and *M. intracellulare*, constitutes the predominant cause of nontuberculous mycobacterial pulmonary disease (NTM-PD) in Korea, followed by the *M. abscessus* complex. Its global prevalence is increasing, as shown by a marked rise in Korea from 11.4 to 56.7 per 100,000 individuals between 2010 and 2021, surpassing the incidence of tuberculosis. Among the older adult population (aged ≥65 years), the prevalence escalated from 41.9 to 163.1 per 100,000, accounting for 47.6% of cases by 2021. Treatment should be individualized based on prognostic indicators, including cavitary disease, low body mass index, and positive sputum smears for acid-fast bacilli. Current therapeutic guidelines recommend a 3-drug regimen—consisting of a macrolide, rifampin, and ethambutol—administered for a minimum of 12 months following culture conversion. Nevertheless, treatment success rates are only roughly 60%, and over 30% of patients experience recurrence. This is often attributable to reinfection rather than relapse. Antimicrobial susceptibility testing for clarithromycin and amikacin is essential, as resistance significantly worsens prognosis. Ethambutol plays a crucial role in preventing the development of macrolide resistance, whereas the inclusion of rifampin remains a subject of ongoing debate. Emerging therapeutic strategies suggest daily dosing for milder cases, increased azithromycin dosing, and the substitution of rifampin with clofazimine in severe presentations. Surgical resection achieves a notable sputum conversion rate of approximately 93% in eligible candidates. For refractory MAC-PD, adjunctive therapy with amikacin is advised, coupled with strategies to reduce environmental exposure. Despite advancements in therapeutic approaches, patient outcomes remain suboptimal, highlighting the urgent need for novel interventions.

## Introduction

### Background

Nontuberculous mycobacteria (NTM) are ubiquitous organisms found in various environments, including soil and water [1]. Although NTM infections can affect both pulmonary and extrapulmonary systems, pulmonary disease (PD) is the most common clinical manifestation worldwide [2]. The global prevalence and incidence of NTM-PD have steadily increased, with *Mycobacterium avium* complex (MAC) being the leading cause, followed by the *M. abscessus* complex [3,4].

In Korea, the annual prevalence of NTM-PD increased from 11.4 per 100,000 in 2010 to 56.7 per 100,000 in 2021, surpassing the tuberculosis (TB) rate of 52.1 per 100,000 in 2021 [5]. Notably, among older adults (aged ≥65 years), the prevalence rose dramatically from 41.9 to 163.1 per 100,000, with this age group accounting for 47.6% of total cases in 2021 [5]. Given the rapidly aging demographic structure in Korea, the proportion of older adults with NTM-PD is expected to increase, which will impose a substantial socioeconomic burden on the national healthcare system [5].

The most significant human pathogens in MAC are *M. avium*, *M. intracellulare*, and *M. chimaera*. Although MAC includes several other species, such as *M. arosiense*, *M. colombiense*, *M. bouchedurhonense*, *M. marseillense*, *M. vulneris*, *M. timonense*, and *M. yongonense*, most laboratories are unable to identify these species and subspecies due to the lack of capacity to conduct the molecular methods necessary for their detection [3]. In Korea, the most common causative species of NTM-PD are MAC (79.8%), comprising *M. avium* (41.4%) and *M. intracellulare* (38.4%), followed by the *M. abscessus* complex (16.4%) [4]. Although the number of reported NTM-PD cases has risen over the past decades, the proportions of causative species have remained largely unchanged [4].

Treating NTM-PD is challenging. Even with more than 12 months of treatment using at least 3 antibiotics, overall success rates for MAC-PD hover around 60% [6-10]. Furthermore, even after successful treatment, more than one-third of patients experience recurrence of MAC-PD [11]. Thus, current treatment strategies for MAC-PD must be reassessed to improve clinical outcomes.

### Objectives

This review comprehensively summarizes existing guidelines and recent updates in treatment approaches for MAC-PD.

## Ethics statement

As this study is a literature review, it did not require institutional review board approval or individual consent.

## Clinical presentations of MAC-PD

The diagnostic criteria for NTM-PD were established in the 2007 American Thoracic Society, and Infectious Diseases Society of America (ATS/IDSA) guidelines and have remained unchanged in the 2020 American Thoracic Society, European Respiratory Society, European Society of Clinical Microbiology and Infectious Diseases, and Infectious Diseases Society of America (ATS/ERS/ESCMID/IDSA) guidelines. To diagnose NTM-PD, all clinical, radiologic, and microbiologic criteria should be met [2,12] (Table 1).

The natural course of NTM-PD is influenced by its clinical presentation, which can be categorized into 2 types: fibrocavitary and nodular bronchiectatic [2,3]. Traditionally, the fibrocavitary type is the most common. It is characterized by cavitary lesions in the upper lobes and is often associated with other pulmonary conditions, such as a history of TB or chronic obstructive pulmonary disease. This form typically develops in older males with a history of cigarette smoking and/or alcohol abuse. If left untreated, it rapidly progresses within 1–2 years, potentially leading to extensive lung destruction and respiratory failure [2,3].

In contrast, the nodular bronchiectatic type typically presents as bilateral bronchiectasis with nodular opacities and/or centrilobular infiltrates, frequently affecting the right middle lobe and/or lingula segment. This type predominantly develops in postmenopausal nonsmoking females and usually progresses more slowly than the fibrocavitary form [2,3]. In advanced stages, even this type may develop cavitary lesions [13,14].

A significant proportion of patients with NTM-PD have underlying lung diseases, such as bronchiectasis or TB-destroyed lungs, which raises concerns about bacterial coinfections [15]. Recent studies have reported the clinical features of patients with NTM-PD and bacterial coinfections. Among 180 patients who underwent bronchoscopy, 169 (93.9%) had bronchiectasis and 22 (12.2%) had TB-destroyed lungs. MAC was identified in 153 patients (85.0%), and bacterial coinfections were present in 80 individuals (44.4%). The most commonly identified bacteria were *Klebsiella pneumoniae* (25/80, 31.3%), followed by *Pseudomonas aeruginosa* (20/80, 25.0%) and *Staphylococcus aureus* (20/80, 25.0%). Compared with those without *P. aeruginosa*, patients with this bacterium were older, had a higher prevalence of smoking, exhibited more respiratory symptoms such as cough, and showed more extensive lung involvement [15].

## Natural courses and treatment decision of MAC-PD

The diagnosis of MAC-PD does not always require immediate treatment. Approximately 40%–60% of patients remain stable for several years after diagnosis without intervention [14,16]. Furthermore, around 40%–50% of patients achieve spontaneous negative culture conversion without therapy [16,17]. Therefore, physicians should closely monitor disease progression and make timely decisions about initiating treatment, weighing the potential risks and benefits.

Observational studies have identified several prognostic factors linked to the progression and mortality of MAC-PD. These include: (1) cavitary lesions [14,16,18], (2) low body mass index (BMI) [16-18], (3) extensive disease [16,19], and (4) positive sputum acid-fast bacilli (AFB) smear [16,17]. Treatment should be considered when patients exhibit these risk factors.

A scoring system, known as the BACES (BMI, age, cavity, erythrocyte sedimentation rate, and sex) score, was recently developed to predict mortality in patients with NTM-PD [20]. The BACES score comprises 5 items: (1) BMI <18.5 kg/m^2^, (2) age ≥65 years, (3) presence of a cavity, (4) elevated erythrocyte sedimentation rate, and (5) male sex (each item is assigned one point). The estimated risk of 5-year mortality increases with higher BACES scores, ranging from 1.2% at a score of 0 to 82.9% at a score of 5, demonstrating excellent discrimination performance (Harrell’s C index=0.812) [20]. A higher BACES score is associated with greater disease severity (e.g., positive sputum AFB smear and cavity presence), an increased risk of disease progression, persistent sputum AFB smear positivity, and higher all-cause and disease-specific mortality [21]. Although the BACES score was not designed to predict treatment response in MAC-PD, it may help guide decisions on whether patients should be monitored or require immediate treatment (scores 0–1: observation; scores 2–3: treatment if symptomatic; scores 4–5: immediate treatment) [20] (Table 2).

## Current guidelines for antibiotics treatment

Although some randomized controlled trials have investigated 3-drug regimens that include a macrolide, no well-designed landmark study has definitively established this combination therapy for MAC-PD [22-24]. Recent systematic reviews have reported improved sputum culture conversion rates (54% vs. 38%) and overall treatment success (65.7%) with macrolide-containing regimens [8,9]. Moreover, in cases of macrolide-resistant disease, sputum culture conversion rates drop to approximately 20% [25]. Thus, macrolides remain a crucial component of MAC-PD treatment because outcomes are poor without them. Regarding companion drugs, only regimens that combine rifampin with ethambutol or clofazimine with ethambutol have been shown to prevent macrolide resistance during treatment [22,26,27]. Ethambutol is the most effective companion drug in this regard. Although the role of rifampin is not entirely clear, current guidelines favor its use until further evidence shows that macrolide resistance develops similarly in both 3-drug and 2-drug regimens [12].

The 2020 ATS/ERS/ESCMID/IDSA guidelines recommend a 3-drug macrolide-based regimen for patients with macrolide-susceptible MAC-PD. This regimen includes a macrolide (azithromycin or clarithromycin), rifampin, and ethambutol [12]. Since the 1997 ATS guidelines, this 3-drug regimen has been a cornerstone of MAC-PD treatment and has served as the basis for many subsequent studies [28]. For patients with advanced or severe bronchiectatic, cavitary, or macrolide-resistant MAC-PD, parenteral amikacin (or streptomycin) should be added to the initial treatment for at least 2–3 months [12]. Dosing frequency varies among disease types [12]; an intermittent regimen (3 times per week) is recommended for patients with the nodular bronchiectatic type, whereas a daily regimen is advised for those with cavitary or severe bronchiectatic disease. Treatment should continue for at least 12 months after sputum culture conversion. If culture conversion is not achieved after 6 months of guideline-based treatment, indicating refractory disease, amikacin liposome inhalational suspension (ALIS) is recommended [12,29].

The 2017 British Thoracic Society (BTS) guidelines recommend a similar regimen consisting of a macrolide, rifampin, and ethambutol [30]. Although both guidelines propose intermittent and daily regimens, their indications differ slightly. The BTS guidelines recommend a daily regimen with parenteral or nebulized amikacin for patients with severe MAC-PD, defined by a positive sputum AFB smear, lung cavitation or severe disease, marked systemic symptoms, or a history of treatment failure [30]. Thus, a daily regimen is not limited solely to cavitary disease.

Several discrepancies exist between the 2 guidelines regarding drug regimens. First, the BTS guidelines recommend azithromycin 250 mg daily for a daily regimen, while the ATS/ERS/ESCMID/IDSA guidelines suggest 250–500 mg daily. Second, the BTS guidelines propose parenteral aminoglycosides for up to 3 months for severe MAC-PD, whereas the ATS/ERS/ESCMID/IDSA guidelines recommend aminoglycosides for at least 2–3 months for cavitary or advanced/severe bronchiectatic MAC-PD. Third, although both guidelines advocate the use of parenteral aminoglycosides for advanced/severe bronchiectatic MAC-PD, the ATS/ERS/ESCMID/IDSA guidelines do not clarify whether a daily or intermittent regimen should be used, while the BTS guidelines favor a daily regimen. Fourth, the BTS guidelines recommend using a parenteral formulation of amikacin for nebulization, whereas the ATS/ERS/ESCMID/IDSA guidelines recommend ALIS (Table 3).

## Treatment outcomes of MAC-PD

Over the past decade, several systematic reviews and meta-analyses have examined the treatment outcomes of MAC-PD [6-10]. Success rates varied across studies due to differences in study design, drug regimens, treatment outcomes, and disease severity. Pooled success rates ranged from 39% to 68%, which remains relatively lower than those for TB [6-10]. Subgroup analyses consistently showed that macrolide-containing regimens yielded better outcomes than those without macrolides [6-8,10]. Furthermore, the ATS-recommended 3-drug regimen (macrolide, rifampin, and ethambutol) demonstrated superior outcomes (61.4% success compared to 52.3% for other macrolide-containing regimens), with even more favorable results when maintained for at least 1 year (65.7%) [9] (Table 4).

## Antimicrobial susceptibility testing

Before initiating treatment, it is essential to perform antimicrobial susceptibility testing for clarithromycin (a representative macrolide) and amikacin, as *in vitro* susceptibility for these drugs correlates with treatment outcomes [12]. The resistance breakpoints are defined as ≥32 µg/mL for clarithromycin, >64 µg/mL for parenteral amikacin, and ≥128 µg/mL for ALIS [12,31]. Although susceptibility testing can be conducted for other drugs, their clinical utility remains uncertain. For example, Luo et al. [32] proposed tentative epidemiological cutoff values for clofazimine in *M. avium* and *M. intracellulare* as 1 μg/mL and 2 μg/mL, respectively; however, further studies are needed to confirm these breakpoints.

A recent report from a referral hospital in Korea (2016–2020) detailed antimicrobial susceptibility patterns among NTM isolates [31]. Among 308 strains of MAC, nearly all were susceptible to clarithromycin (*M. avium*: 90/91, 99%; *M. intracellulare*: 217/217, 100%). However, susceptibility to amikacin was slightly lower in *M. avium* (69/91, 76%) than in *M. intracellulare* (191/217, 88%; P=0.01) [31]. Disappointingly, most MAC strains were not susceptible to moxifloxacin (*M. avium*: 20/91, 22%; *M. intracellulare*: 18/217, 8%). and linezolid (*M. avium*: 27/91, 30%; *M. intracellulare*: 16/217, 7%) [31]. These data underscore the crucial role of macrolide in MAC treatment.

## Constructing treatment regimen

### Macrolides

Among macrolides, azithromycin is generally preferred over clarithromycin in most clinical settings. This preference is due to its once-daily dosing (compared to clarithromycin’s twice-daily schedule), fewer side effects and drug interactions, and a serum concentration that is less affected by coadministration with rifampin [12,33]. Gastrointestinal disturbances are common with long-term macrolide use and occur more frequently with clarithromycin than with azithromycin.

For mild disease, an intermittent regimen is often preferred over a daily regimen because it is associated with fewer gastrointestinal disturbances and yields comparable treatment outcomes, as previous studies have demonstrated [12,34]. A meta-analysis reported similar treatment success rates between intermittent and daily regimens (61%; 95% confidence interval [CI], 55%–67% vs. 60%; 95% CI, 53%–66%) [7]. However, one landmark study found that overall treatment outcomes were not entirely satisfactory: symptom improvement occurred in 75% of patients on daily treatment compared to 82% on intermittent treatment (P=0.181), radiologic improvement was observed in 68% vs. 73% (P=0.402), and sputum culture conversion rates were 76% vs. 67% (P=0.154) [34].

A recent report evaluated daily regimen outcomes in non-cavitary nodular bronchiectatic MAC-PD [35]. Among 110 patients, 53 (48.2%) received daily treatment. The culture conversion rate was significantly higher in the daily group than in the intermittent group (90.6% [48/53] vs. 70.2% [40/57], P=0.008). This difference was particularly notable in 36 patients with a positive AFB smear, where conversion rates were 85.0% (17/20) for daily treatment vs. 50.0% (8/16) for intermittent treatment (P=0.034). Even among patients with a negative AFB smear, the daily group achieved a higher conversion rate (93.9% [31/33] vs. 78.0% [32/41], P=0.098) [35]. These findings suggest that a daily regimen for non-cavitary nodular bronchiectatic MAC-PD may improve outcomes—a recommendation also supported by the 2017 BTS guidelines [30].

Current guidelines recommend an azithromycin dose of 250 mg daily or 500 mg 3 times weekly [12,30]. However, the peak serum concentration (C_max_) of azithromycin was found to be lower with a daily regimen than with an intermittent regimen (median: 0.24 μg/mL vs. 0.65 μg/mL, P<0.001), as rifampin may significantly reduce the C_max_ of azithromycin in the daily regimen [36]. In the daily regimen, a lower azithromycin C_max_ was common, whereas a higher azithromycin C_max_ was linked to favorable microbiologic responses [36]. For severe MAC-PD, such as cavitary or smear-positive disease, the currently recommended azithromycin dose might be suboptimal, and at least 500 mg daily should be used.

Macrolide monotherapy should be avoided in MAC-PD because it can lead to macrolide resistance [12,30,37]. Treatment outcomes in patients with macrolide-resistant MAC-PD are poor; sputum culture conversion following antibiotic treatment and surgical resection reaches only 21% (95% CI, 14%–30%), with 1-year all-cause mortality at 10% (95% CI, 5%–20%) [25]. Thus, long-term macrolide maintenance therapy should be selected cautiously to prevent exacerbations of bronchiectasis or cystic fibrosis [37]. Given the poor treatment outcomes in cases of macrolide-resistant MAC, preserving macrolide susceptibility is a critical aspect of MAC treatment strategies.

### Ethambutol

Ethambutol is the second most important antibiotic in the treatment of MAC-PD, as it is the most effective agent identified to date for preventing macrolide resistance [12,30]. It is generally well tolerated; however, its major side effect is optic neuritis, which is typically reversible upon discontinuation [38]. All patients should undergo a baseline ophthalmologic evaluation before treatment initiation and be monitored for symptoms during therapy [12,38]. Dyschromatopsia—an early sign of optic neuritis—can be detected using the Ishihara pseudo-isochromatic plate examination [12,38]. In Korea, the Han pseudo-isochromatic plate is also commonly used. If new visual symptoms develop, ethambutol should be discontinued until an ophthalmologist can rule out ethambutol-related optic toxicity. If an ophthalmologic evaluation is delayed for more than one month, it is reasonable to halt the entire treatment rather than stopping ethambutol alone, to avoid the development of macrolide resistance.

### Rifampin

Rifampin is the third agent in the standard 3‐drug regimen [12,30]. Although current guidelines favor a 3‐drug regimen over a 2‐drug combination (macrolide and ethambutol), this recommendation is conditional and based on very low certainty [12]. The precise role of rifampin in MAC treatment remains uncertain; a more significant concern is its drug interactions, particularly with macrolides [33]. Rifampin induces cytochrome P450 enzymes, thereby lowering the serum concentrations of both macrolides and ethambutol. Concurrent administration of rifampin has been shown to reduce the C_max_ of clarithromycin by 68% and that of azithromycin by 23%, resulting in C_max_ levels falling below the target range in 56% of patients for clarithromycin, 35% for azithromycin, and 48% for ethambutol [33]. Additionally, an *in vitro* hollow fiber experiment comparing the pharmacokinetic profiles of a 3‐drug regimen (azithromycin, ethambutol, and rifampin) with those of a 2‐drug regimen (azithromycin and ethambutol) found that rifampin neither enhanced the antimycobacterial effect nor prevented macrolide resistance [39].

The minimal inhibitory concentration (MIC) of rifampin against *M. avium* (median 4 mg/L) is higher than the current clinical breakpoints for M. tuberculosis (0.5 mg/L). A target area under the time–concentration curve (AUC)/MIC ratio of >197.3 was reported as driving the efficacy of rifampin against MAC. Given the previously reported rifampin mean AUC of 68.42 mg·hr/L in patients with MAC-PD, the median MIC of 4 mg/L requires AUCs as high as 789.2 (197.3×4) mg·hr/L for rifampin to be effective. Even in patients with TB receiving rifampin at 50 mg/kg, the mean AUC only reaches 571 mg·hr/L [40]. These findings cast doubt on the effectiveness of rifampin in NTM treatment and may help explain the unsatisfactory outcomes associated with current treatment guidelines.

Several studies have suggested that a 2‐drug regimen consisting of a macrolide and ethambutol may be as effective as a 3‐drug regimen that includes rifampin. For instance, a randomized controlled trial in Japan compared a 3‐drug regimen (clarithromycin, ethambutol, and rifampicin) with a 2‐drug regimen (clarithromycin and ethambutol) in patients with MAC-PD [22]. Among 119 patients, culture conversion was achieved in 40.6% (24/59) of those receiving the 3‐drug regimen and in 55.0% (33/60) of those receiving the 2‐drug regimen. Even in patients with cavitary disease, culture conversion rates were similar between the 2 groups, with 73.3% (11/15) for the 3‐drug regimen and 68.7% (11/16) for the 2‐drug regimen [22].

A retrospective study in Korea examined the efficacy of an intermittent regimen of azithromycin and ethambutol for non‐cavitary MAC-PD [41]. In this study of 38 patients, 29 (76%) achieved culture conversion after 12 months of treatment, and notably, none of the 9 patients who did not convert developed macrolide resistance. A positive AFB smear was significantly associated with treatment failure (adjusted odds ratio [aOR], 26.7; 95% CI, 2.1–339.9) [41].

A third retrospective study from Korea compared the individual contributions of rifampin and ethambutol in MAC-PD [42]. In this study of 237 patients, 122 (51.5%) received a regimen of macrolide, ethambutol, and rifampin; 58 (24.5%) received macrolide and ethambutol; 32 (13.5%) received macrolide and rifampin; and 25 (10.6%) received macrolide alone. Overall, 190 of 237 patients (80.2%) achieved culture conversion after a median treatment duration of 1.7 months (interquartile range [IQR], 0.5–4.7), and a microbiological cure was observed in 129 of 177 patients (72.9%) who completed treatment. Compared to macrolide monotherapy, combining a macrolide with both ethambutol and rifampin (aOR, 5.12; 95% CI, 1.72–15.24) or with ethambutol alone (aOR, 5.74; 95% CI, 1.54–21.42) was significantly associated with microbiological cure, whereas combining macrolide with rifampin was not (aOR, 2.43; 95% CI, 0.69–8.58) [42]. These results call into question the additional benefit of including rifampin in a regimen that already contains macrolide and ethambutol.

For MAC-PD cases with a low mycobacterial burden—such as non-cavitary, smear-negative disease—a 2-drug regimen of azithromycin and ethambutol may be sufficient. Conversely, for MAC-PD with a high mycobacterial burden, such as cavitary or smear-positive disease, a 3-drug regimen might be necessary; however, the third agent should likely not be rifampin.

### Clofazimine

Recently, clofazimine, which was originally used to treat leprosy, has been repurposed for the treatment of NTM-PD [12,30]. Although several studies have demonstrated its efficacy in MAC-PD, clofazimine has not yet been fully incorporated into current guidelines because these studies were published after the guidelines were issued. For example, a retrospective study conducted in Canada evaluated treatment outcomes of a clofazimine-containing regimen [26]. Among 107 patients, 90 (84%) received a regimen of clofazimine, macrolide, and ethambutol, while 14 (13%) received a regimen of rifampin, macrolide, and ethambutol. All 90 patients (100%) treated with clofazimine achieved sputum culture conversion, compared to 71% (10/14, P=0.0002) in the rifampin group. Microbiological relapse occurred in 52 of 107 patients (49%), with no significant difference between the 2 groups [26].

Another retrospective study from Korea assessed the outcomes of a clofazimine-containing regimen in severe MAC-PD [43]. Among 170 patients, 121 (71.2%) had cavitary disease. All patients received a macrolide, and 150 (88.2%) received ethambutol (excluding 20 patients with prior ophthalmic complications); only 15 (8.8%) received rifampicin. Within 6 months, 77 patients (45.3%) achieved culture conversion, and microbiological cures were observed in 84 of 154 patients (54.6%). Notably, the microbiological cure rate increased to 71.0% (22/31) among patients who received clofazimine for 6–12 months, compared to 23.1% (6/26) in those treated for less than 6 months [43].

Finally, a randomized controlled trial conducted in the Netherlands compared regimens in which 21 patients received clofazimine and 19 received rifampin, both in addition to macrolide and ethambutol [44]. Sputum culture conversion rates were similar in both groups, with 62% (13/21) in the clofazimine group and 58% (11/19) in the rifampin group. Moreover, pharmacokinetic data revealed that the maximum serum concentration (C_highest_) of azithromycin was significantly higher in the clofazimine group compared to the rifampin group (0.64 mg/L vs. 0.35 mg/L at 1 month, P=0.005; 0.75 mg/L vs. 0.31 mg/L after 4 months, P<0.001) [44]. These findings support the inclusion of clofazimine in MAC-PD treatment regimens.

Currently, clofazimine is administered at a daily dose of 100 mg; however, its pharmacokinetics are complex due to its high protein-binding, lipophilicity, and accumulation in adipose tissue, which results in an extremely long elimination half-life of 30–70 days [45]. Consequently, it takes several months for plasma concentrations to reach steady state. In an *in vivo* experiment involving 12 individuals, the C_max_ of clofazimine was 0.87 mg/L after a loading dose of 300 mg daily for 4 weeks, with adverse events remaining tolerable. In a virtual simulation of 1,000 individuals, the median time to reach the target concentration (defined as 80% of steady state) was 5.3 months with a 100 mg daily regimen without a loading dose. However, when a loading dose of 300 mg daily was administered for 4 weeks followed by maintenance at 100 mg daily, the median time to target concentration decreased to 3.8 months. Extending the loading phase to 6 weeks further reduced the median time to 1.4 months [45]. These results suggest that incorporating a loading phase of clofazimine should be considered to accelerate the attainment of steady-state concentrations.

### Amikacin

Parenteral aminoglycosides, such as amikacin and streptomycin, are recommended for patients with cavitary or severe MAC-PD [12,30]. A retrospective study in Korea compared treatment outcomes in cavitary MAC-PD between patients who received streptomycin and those who received amikacin [46]. Among 168 patients treated with a guideline-based 3-drug regimen plus a parenteral aminoglycoside, 127 (75.6%) received streptomycin and 41 (24.4%) received amikacin for a median duration of 17.1 weeks (IQR, 14.0–17.6 weeks). The overall culture conversion rate was 75.6% (127/168), with similar rates observed in the streptomycin group (74.8%, 95/127) and the amikacin group (78.0%, 32/41; P=0.674). Adverse event rates were comparable between the 2 groups [46]. These findings indicate that either amikacin or streptomycin can be effectively used in the treatment of MAC-PD.

Systemic administration of aminoglycosides is associated with serious adverse effects, including ototoxicity and renal toxicity, which limit their long-term use. Consequently, inhaled amikacin has emerged as an alternative that maintains efficacy while reducing systemic toxicity. The 2020 ATS/ERS/ESCMID/IDSA guidelines recommend ALIS for patients with cavitary or refractory MAC-PD, a recommendation supported by a phase 3 randomized controlled trial [12]. However, ALIS carries a high economic burden (approximately US$16,000 per 4 weeks) and is not covered by the national health insurance program in Korea, limiting its widespread adoption [29]. Moreover, treatment outcomes with ALIS in Korea have been unsatisfactory; among 6 patients with refractory MAC-PD treated with ALIS, one was already culture-negative before its initiation, and only one of the remaining 5 patients with positive cultures achieved culture conversion [29].

Although the 2017 BTS guidelines recommended the inhalation of a parenteral formulation of amikacin, the supporting evidence is limited. A recent retrospective study investigated the efficacy of inhaled amikacin in combination with clofazimine in refractory MAC-PD [47]. In this study of 52 patients, 17 (33%) achieved culture conversion, and only 12 (23%) attained microbiological cure [47]. These findings suggest that while inhaled amikacin with clofazimine may offer favorable outcomes in some cases of refractory MAC-PD, more effective treatment strategies are still needed.

### Reevaluating treatment strategies beyond guidelines

Recent research suggests several novel treatment strategies that warrant consideration: (1) employing a daily treatment regimen for non-severe MAC-PD; (2) using a daily dose of 500 mg of azithromycin in such regimens; (3) omitting rifampin in non-severe MAC-PD; (4) replacing rifampin with clofazimine in severe MAC-PD; and (5) incorporating a loading phase for clofazimine. These strategies are summarized in Table 5.

## Refractory and recurrent MAC-PD

Despite long-term antibiotic treatment, overall success rates for MAC-PD remain unsatisfactory, with a significant proportion of patients remaining culture-positive, indicating refractory disease [6-10]. Interestingly, macrolide resistance is uncommon among patients with refractory MAC-PD who are on long-term macrolide-containing regimens [34]. One study demonstrated that refractory MAC-PD is usually due to reinfection with new strains rather than persistent infection [48]. In a cohort of 481 treatment-naive MAC-PD patients, 72 (15.0%) remained sputum culture-positive after at least 12 months of treatment and were classified as having refractory disease. Among the 49 patients with both pre- and post-treatment isolates, mycobacterial genotyping revealed that 36 (73%) had been reinfected with new strains, while only 13 (27%) had persistent infection with their original strains [48]. These findings may explain why macrolide resistance is rare in refractory MAC-PD.

Recurrences of MAC-PD are also common following the completion of long-term antibiotic therapy. However, evidence suggests that most recurrences represent reinfections rather than treatment failures or relapses [13,49]. For example, one study reported that an intermittent regimen (3 times weekly) in patients with recurrent non-cavitary nodular bronchiectatic MAC-PD achieved similar sputum culture conversion rates compared to a daily regimen (82% [22/27] vs. 81% [21/26]) [49]. Additionally, in 15 of 53 patients (28%), different MAC species were identified compared to the previous treatment, and among 38 patients with the same species, genotype analysis revealed that 86% (12/14) of cases were reinfections with new strains [49].

Current guidelines recommend the adjunctive use of parenteral or nebulized amikacin alongside a 3-drug oral regimen to reinforce treatment in refractory or recurrent MAC-PD [12,30]. However, given that a substantial proportion of these cases are due to reinfection, adjunctive amikacin may be unnecessary and could cause undesirable adverse effects, particularly in non-severe cases (e.g., negative sputum AFB smear, bronchiectatic disease without cavitation, or mild-to-moderate symptoms) [30]. In such cases, oral regimens might be feasible for selected patients, depending on disease severity and microbiologic evidence. Moreover, to reduce the risk of reinfection from environmental exposures, lifestyle and environmental modifications should be recommended for these patients [50].

There is currently no proven treatment strategy for macrolide-resistant MAC-PD. While maintenance of macrolide therapy is common after the detection of macrolide resistance, surgical resection and prolonged parenteral aminoglycoside administration remain crucial treatment strategies [25]. The 2017 BTS guidelines suggest adding other drugs, such as isoniazid, moxifloxacin, or nebulized amikacin, although the efficacy of these regimens remains inconclusive [30]. Recently, bedaquiline and ALIS have been investigated for the treatment of macrolide-resistant MAC-PD; however, treatment outcomes have been unsatisfactory [51,52].

## Surgical treatment

The success rate of medical treatment for MAC-PD has been unsatisfactory, with many patients experiencing treatment failure due to macrolide resistance. Consequently, adjuvant surgical lung resection has been employed to improve outcomes in selected patients [3,12,30,53]. Typically, patients with focal parenchymal disease and sufficient predicted pulmonary reserve following surgery are considered candidates for resection; unfortunately, only a minority of NTM-PD patients meet these criteria [53].

There are 3 main indications for surgery [53]. First, surgical treatment is usually considered after the failure of medical therapy, particularly when the disease is confined to a focal parenchymal region while the remainder of the lung is relatively unaffected. Surgical resection may also be indicated in cases of recurrent treatment failures, antimicrobial resistance, or intolerance to antimicrobial agents. Second, surgery may be performed to alleviate or eliminate potentially life-threatening symptoms, such as hemoptysis; resection can control these symptoms even if some residual disease remains. Third, in a small subset of cases, debulking surgery (i.e., removal of the most severely damaged lung parenchyma) may slow disease progression by limiting the spread of infection to less affected areas [53].

A recent meta-analysis synthesized results from 15 studies involving 1,071 patients with NTM-PD who underwent adjunctive surgical treatment [54]. After surgical resection, sputum culture conversion was achieved in 93% (95% CI, 87%–97%), and recurrence was identified in 9% (95% CI, 6%–14%) of patients during a median 34 months of follow-up. Additionally, 17% (95% CI, 13%-23%) experienced postoperative complications, and in-hospital mortality occurred in 0% (95% CI, 0%–2%) [54]. These findings suggest that adjunctive surgical treatment is an effective therapeutic alternative with acceptable complication rates.

## Nonpharmacological treatment

NTM are ubiquitous environmental pathogens commonly found in natural water, soil, and household environments [1,50]. Water distribution systems, such as plumbing, are key transmission routes into households. NTM have been isolated from drinking water tanks and pipelines, where they adhere to surfaces and form biofilms due to their lipid-rich, hydrophobic outer membrane. This membrane also confers natural resistance to disinfectants like chlorine, making water chlorination a more favorable environment for NTM survival compared to other microorganisms [1].

Several strategies are recommended to reduce exposure to environmental water: (1) heat water to temperatures exceeding 55–65°C before use; (2) use bacteriologic filters (with pore sizes <0.45 μm) on showerheads and taps; (3) regularly clean showerheads with vinegar to remove biofilms; (4) choose showerheads with larger holes to reduce aerosol formation; (5) shorten the duration of showers; (6) avoid spas or hot tubs; and (7) boil drinking water for 10 minutes (noting that bottled water is not free from NTM contamination) [1,50].

Household dust and soil also serve as significant sources of NTM exposure. Soil particles can become aerosolized during activities like farming or gardening, increasing the risk of inhalation. To reduce exposure, it is recommended to moisten soil before handling it or to wear masks during these activities [1,50].

In addition to minimizing environmental exposure, it is important to address host factors that increase vulnerability to NTM-PD [50]. First, impaired mucus clearance from the airways is a significant risk factor; therefore, hydration and airway clearance techniques, such as using mechanical oscillation devices (e.g., oscillating positive expiratory pressure devices), are recommended. Second, a lower BMI is associated with a higher incidence of NTM-PD and a poorer prognosis, so increasing caloric intake and protein supplementation may be beneficial. Third, gastroesophageal reflux disease is linked to an increased risk of NTM-PD (subdistribution hazard ratio, 3.36; 95% CI, 2.10–5.37) [55]. It is hypothesized that NTM may enter the stomach via drinking water and then be aspirated into the lungs along with gastric acid reflux [50]. The inherent acid resistance of NTM, due to their lipid-rich outer membrane, further contributes to this mechanism [1]. To manage gastroesophageal reflux disease, dietary modifications (such as avoiding coffee, alcohol, and carbonated beverages), postural adjustments (such as elevating the head during sleep and remaining upright after meals), and smoking cessation are recommended (Table 6) [50].

## Conclusion

Although the prevalence of MAC-PD has been rapidly increasing, the treatment outcomes of current guideline-based regimens remain unsatisfactory. To improve these outcomes, treatment strategies must be updated in light of recent research findings. Since 2020, a multicenter prospective observational cohort study (NTM-KOREA) has been launched to optimize treatment modalities for NTM-PD in Korea [56]. We hope that further investigation under this program will lead to more effective treatment strategies for MAC-PD.

## Figures and Tables

**Table 1.** Diagnostic criteria for nontuberculous mycobacterial pulmonary disease

	Diagnostic criteria
Clinical	(1) Pulmonary or systemic symptoms and (2) appropriate exclusion of other diagnoses
Radiologic	(1) Nodular or cavitary opacities on chest radiograph or (2) bronchiectasis with multiple small nodules on high-resolution computed tomography scan
Microbiologic	(1) Positive culture results from at least 2 separate expectorated sputum samples (over an interval of a week or more) or (2) positive culture results from at least one bronchial wash or lavage or (3) transbronchial or other lung biopsy with mycobacterial histologic features (granulomatous inflammation or AFB) and positive culture for NTM or biopsy showing mycobacterial histologic features (granulomatous inflammation or AFB) and one or more sputum or bronchial washings that are culture positive for NTM
	When 2 positive cultures are obtained, the isolates should be the same NTM species (or subspecies in the case of *Mycobacterium abscessus*) in order to meet disease criteria.

AFB, acid-fast bacilli; NTM, nontuberculous mycobacteria.

**Table 2.** The BACES score system

Variable	Detail	Point
Body mass index (kg/m^2^)	<18.5	1
Age (yr)	≥65	1
Cavity	Visible on chest computed tomography	1
Elevated erythrocyte sedimentation rate	>15 mm/hr for male, >20 mm/hr for female	1
Sex	Male	1
Treatment recommendation		
Scores 0–1	Observation	
Scores 2–3	Treatment if symptomatic	
Scores 4–5	Immediate treatment	

BACES, body mass index, age, cavity, erythrocyte sedimentation rate, and sex.

**Table 3.** Treatment of *Mycobacterium avium* complex pulmonary disease according to current guidelines

Disease types	No. of drugs	Drug regimen	Treatment duration
2020 American Thoracic Society, European Respiratory Society, European Society of Clinical Microbiology and Infectious Diseases, and Infectious Diseases Society of America guidelines [12]			
Nodular-bronchiectatic MAC-PD	3	• Azithromycin 500 mg TIW (clarithromycin 1 g in 2 divided doses TIW)	At least 12 months after culture conversion
		• Rifampin 600 mg TIW (rifabutin 300 mg TIW)	
		• Ethambutol 25 mg/kg TIW	
Cavitary MAC-PD	≥3	• Azithromycin 250–500 mg daily (clarithromycin 500 mg twice daily)	At least 12 months after culture conversion
		• Rifampin 450 mg or 600 mg daily (rifabutin 150–300 mg daily, 150 mg daily with clarithromycin)	
		• Ethambutol 15 mg/kg daily	
		• Intravenous amikacin 10–15 mg/kg daily or 15–25 mg/kg TIW (streptomycin 10–15 mg/kg daily or 15–25 mg/kg TIW) for at least 2–3 months	
Refractory MAC-PD (sputum culture positive after 6 months of guideline-based therapy)	≥4	• Azithromycin 250–500 mg daily (clarithromycin 500 mg twice daily)	At least 12 months after culture conversion
		• Rifampin 450 mg or 600 mg daily (rifabutin 150–300 mg daily, 150 mg daily with clarithromycin)	
		• Ethambutol 15 mg/kg daily	
		• Amikacin liposome inhalational suspension or 590 mg per day	
		• Intravenous amikacin 10–15 mg/kg daily or 15–25 mg/kg TIW (streptomycin 10–15 mg/kg daily or 15–25 mg/kg TIW) for at least 2–3 months	
Macrolide-resistant MAC-PD		• Expert consultation	
2017 British Thoracic Society guidelines [30]			
Non-severe MAC-PD (i.e., negative sputum AFB smear, no radiological evidence of lung cavitation or severe infection, mild-moderate symptoms, no signs of systemic illness)	3	• Azithromycin 500 mg TIW or clarithromycin 1 g in 2 divided doses TIW	A minimum of 12 months after culture conversion
		• Rifampin 600 mg TIW	
		• Ethambutol 25 mg/kg TIW	
Severe MAC-PD (i.e., positive sputum AFB smear, radiological evidence of lung cavitation/severe infection, severe symptoms/signs of systemic illness, or a history of treatment failure)	≥4	• Azithromycin 250 mg daily or clarithromycin 500 mg twice daily	A minimum of 12 months after culture conversion
		• Rifampin 600 mg daily	
		• Ethambutol 15 mg/kg daily	
		• Intravenous amikacin 15 mg/kg once daily or 7.5 mg/kg twice daily or 15–25 mg/kg TIW for up to 3 months or intravenous streptomycin 15 mg/kg daily for initial 1 month, followed by 15 mg/kg TIW for up to 3 months or nebulized amikacin 500 mg twice daily (dose reduction: 250–500 mg once or twice daily)	
Clarithromycin-resistant MAC-PD	≥4	• Rifampin 600 mg daily	A minimum of 12 months after culture conversion
		• Ethambutol 15 mg/kg daily	
		• Isoniazid 300 mg (+pyridoxine 10 mg) daily or moxifloxacin 400 mg daily	
		• Intravenous amikacin 15 mg/kg once daily or 7.5 mg/kg twice daily or 15–25 mg/kg TIW for up to 3 months or intravenous streptomycin 15 mg/kg daily for initial 1 month, followed by 15 mg/kg TIW for up to 3 months or nebulized amikacin 500 mg twice daily (dose reduction: 250–500 mg once or twice daily)	

MAC-PD, *Mycobacterium avium* complex pulmonary disease; TIW, 3 times per week; AFB, acid-fast bacilli.

**Table 4.** Summary of meta-analyses of treatment outcomes of *Mycobacterium avium* complex pulmonary disease

Author	Year	No. of studies	No. of participants	Study designs	Definition of treatment success	Estimated pooled rates of study outcomes, % (95% CI)
Xu et al. [6]	2014	28	2,422	Prospective and retrospective study	Variable (specified by mycobacterial culture endpoints)	• Treatment success: 39% (38%–41%)
						• Macrolide-containing regimens: 42% (40%–44%)
						• Macrolide-free regimens: 28% (24%–32%)
Kwak et al. [7]	2017	16	1,462	Randomized controlled trials, observational studies	12 months of sustained culture negativity	• Treatment success: 60.0% (55.1%–64.8%)
						• Macrolide-free regimens: 53.6% (38.0%–69.3%)
Pasipanodya et al. [8]	2017	21	2,534	Prospective studies, clinical trials, reports from established disease registries	Sputum culture conversion	• Sustained sputum culture conversion
						• Macrolide-containing regimens: 54% (45%–63%)
						• Macrolide-free regimens: 38% (25%–52%)
Diel et al. [9]	2018	42	2,748	Randomized study, prospective study, retrospective study	Sputum culture conversion	• Sputum culture conversion
						• Macrolide-containing regimen: 52.3% (44.7%–59.9%)
						• ATS recommended 3-drug regimen: 61.4% (49.7%–72.5%)
						• ATS recommended 3-drug regimen for at least 1 year: 65.7% (53.3%–77.4%)
Nasiri et al. [10]	2020	45	3,862	Randomized trial, retrospective study	Sputum culture conversion and completion of the planned treatment without relapse	• Treatment success: 68.1% (64.7%–71.4%)
						• Macrolide-containing regimens: 69.0% (65.7%–72.3%)
						• Macrolide-free regimens: 58.5% (38.8%–78.2%)

CI, confidence interval; ATS, American Thoracic Society.

**Table 5.** Treatment strategies of *Mycobacterium avium* complex pulmonary disease reflecting recent research

Disease types	No. of drugs	Drug regimen	Treatment duration
Non-severe MAC-PD (i.e., negative sputum AFB smear, no radiological evidence of lung cavitation or severe infection, mild-moderate symptoms, no signs of systemic illness)	2	• Azithromycin 500 mg daily (250 mg, if intolerable to 500 mg)	A minimum of 12 months after culture conversion
		• Ethambutol 15 mg/kg daily	
Severe MAC-PD (i.e., positive sputum AFB smear, radiological evidence of lung cavitation/severe infection, severe symptoms/signs of systemic illness, or a history of treatment failure)	≥4	• Azithromycin 500 mg daily (250 mg, if intolerable to 500 mg)	A minimum of 12 months after culture conversion
Refractory MAC-PD (sputum culture positive after 6 months of guideline-based therapy)		• Ethambutol 15 mg/kg daily	
		• Clofazimine 100 mg daily (consider 200–300 mg for initial 4–6 weeks for loading phase)	
		• Intravenous amikacin 15 mg/kg daily or 15–25 mg/kg TIW for at least initial 2–3 months, followed by nebulized amikacin 250–500 mg once or twice daily	

MAC-PD, *Mycobacterium avium* complex pulmonary disease; AFB, acid-fast bacilli; TIW, 3 times per week.

**Table 6.** Nonpharmacological treatment strategies of *Mycobacterium avium* complex pulmonary disease

Strategy	Method
Avoid environmental exposures	
Reduce exposure to environmental water	• Heat water at temperatures exceeding 55–65°C before use
	• Use bacteriologic filters on showerheads and taps
	• Regularly clean showerheads with vinegar to remove biofilm
	• Use showerheads with large holes to reduce aerosol formation
	• Shorten the duration of showers
	• Avoid spas or hot tubs
	• Boil drinking water for 10 minutes
Reduce exposure to environmental soil	• Moisten soil with water
	• Wear masks during activities
Lifestyle modifications	
Improve airway clearance	• Airway hydration
	• Airway clearance techniques using mechanical oscillation devices
Nutritional support	• Increase caloric intake
	• Protein supplementation
Management of gastroesophageal reflux disease	• Dietary modifications (e.g., avoiding coffee, alcohol, and carbonated beverages)
	• Postural modifications (e.g., elevating head during sleep, and maintaining upright position after meals)
	• Smoking cessation

## References

[1] Jeon D (2019). Infection source and epidemiology of nontuberculous mycobacterial lung disease. Tuberc Respir Dis (Seoul).

[2] Griffith DE, Aksamit T, Brown-Elliott BA, Catanzaro A, Daley C, Gordin F, Holland SM, Horsburgh R, Huitt G, Iademarco MF, Iseman M, Olivier K, Ruoss S, von Reyn CF, Wallace RJ, Winthrop K, ATS Mycobacterial Diseases Subcommittee, American Thoracic Society, Infectious Disease Society of America (2007). An official ATS/IDSA statement: diagnosis, treatment, and prevention of nontuberculous mycobacterial diseases. Am J Respir Crit Care Med.

[3] Daley CL (2017). Mycobacterium avium complex disease. Microbiol Spectr.

[4] Lee G, Kim S, Chang S, Sohn H, Kang YA, Park Y (2024). Epidemiological characteristics of nontuberculous mycobacterial pulmonary disease in South Korea: a meta-analysis of individual participant data. Tuberc Respir Dis (Seoul).

[5] Kim JY, Kwak N, Yim JJ (2022). The rise in prevalence and related costs of nontuberculous mycobacterial diseases in South Korea, 2010-2021. Open Forum Infect Dis.

[6] Xu HB, Jiang RH, Li L (2014). Treatment outcomes for Mycobacterium avium complex: a systematic review and meta-analysis. Eur J Clin Microbiol Infect Dis.

[7] Kwak N, Park J, Kim E, Lee CH, Han SK, Yim JJ (2017). Treatment outcomes of Mycobacterium avium complex lung disease: a systematic review and meta-analysis. Clin Infect Dis.

[8] Pasipanodya JG, Ogbonna D, Deshpande D, Srivastava S, Gumbo T (2017). Meta-analyses and the evidence base for microbial outcomes in the treatment of pulmonary Mycobacterium avium-intracellulare complex disease. J Antimicrob Chemother.

[9] Diel R, Nienhaus A, Ringshausen FC, Richter E, Welte T, Rabe KF, Loddenkemper R (2018). Microbiologic outcome of interventions against Mycobacterium avium complex pulmonary disease: a systematic review. Chest.

[10] Nasiri MJ, Ebrahimi G, Arefzadeh S, Zamani S, Nikpor Z, Mirsaeidi M (2020). Antibiotic therapy success rate in pulmonary Mycobacterium avium complex: a systematic review and meta-analysis. Expert Rev Anti Infect Ther.

[11] Zo S, Kim H, Kwon OJ, Jhun BW (2022). Antibiotic maintenance and redevelopment of nontuberculous mycobacteria pulmonary disease after treatment of Mycobacterium avium complex pulmonary disease. Microbiol Spectr.

[12] Daley CL, Iaccarino JM, Lange C, Cambau E, Wallace RJ, Andrejak C, Bottger EC, Brozek J, Griffith DE, Guglielmetti L, Huitt GA, Knight SL, Leitman P, Marras TK, Olivier KN, Santin M, Stout JE, Tortoli E, van Ingen J, Wagner D, Winthrop KL (2020). Treatment of nontuberculous mycobacterial pulmonary disease: an official ATS/ERS/ESCMID/IDSA clinical practice guideline. Eur Respir J.

[13] Koh WJ, Moon SM, Kim SY, Woo MA, Kim S, Jhun BW, Park HY, Jeon K, Huh HJ, Ki CS, Lee NY, Chung MJ, Lee KS, Shin SJ, Daley CL, Kim H, Kwon OJ (2017). Outcomes of Mycobacterium avium complex lung disease based on clinical phenotype. Eur Respir J.

[14] Lee G, Lee KS, Moon JW, Koh WJ, Jeong BH, Jeong YJ, Kim HJ, Woo S (2013). Nodular bronchiectatic Mycobacterium avium complex pulmonary disease: natural course on serial computed tomographic scans. Ann Am Thorac Soc.

[15] Moon SM, Cho H, Shin B (2024). Exploring the association of bacterial coinfections with clinical characteristics of patients with nontuberculous mycobacterial pulmonary disease. Tuberc Respir Dis (Seoul).

[16] Hwang JA, Kim S, Jo KW, Shim TS (2017). Natural history of Mycobacterium avium complex lung disease in untreated patients with stable course. Eur Respir J.

[17] Pan SW, Shu CC, Feng JY, Wang JY, Chan YJ, Yu CJ, Su WJ (2017). Microbiological persistence in patients with Mycobacterium avium complex lung disease: the predictors and the impact on radiographic progression. Clin Infect Dis.

[18] Kim SJ, Yoon SH, Choi SM, Lee J, Lee CH, Han SK, Yim JJ (2017). Characteristics associated with progression in patients with of nontuberculous mycobacterial lung disease: a prospective cohort study. BMC Pulm Med.

[19] Kim SJ, Park J, Lee H, Lee YJ, Park JS, Cho YJ, Yoon HI, Lee CT, Lee JH (2014). Risk factors for deterioration of nodular bronchiectatic Mycobacterium avium complex lung disease. Int J Tuberc Lung Dis.

[20] Kim HJ, Kwak N, Hong H, Kang N, Im Y, Jhun BW, Yim JJ (2021). BACES score for predicting mortality in nontuberculous mycobacterial pulmonary disease. Am J Respir Crit Care Med.

[21] Kim HJ, Song MJ, Kwon BS, Kim YW, Lim SY, Lee YJ, Park JS, Cho YJ, Lee CT, Lee JH (2023). Usefulness of the BACES score in nontuberculous mycobacterial pulmonary disease for various clinical outcomes. Sci Rep.

[22] Miwa S, Shirai M, Toyoshima M, Shirai T, Yasuda K, Yokomura K, Yamada T, Masuda M, Inui N, Chida K, Suda T, Hayakawa H (2014). Efficacy of clarithromycin and ethambutol for Mycobacterium avium complex pulmonary disease: a preliminary study. Ann Am Thorac Soc.

[23] Fujita M, Kajiki A, Tao Y, Miyazaki M, Ouchi H, Harada E, Ikegame S, Matsumoto T, Uchino J, Watanabe K, Nakanishi Y (2012). The clinical efficacy and safety of a fluoroquinolone-containing regimen for pulmonary MAC disease. J Infect Chemother.

[24] Jenkins PA, Campbell IA, Banks J, Gelder CM, Prescott RJ, Smith AP (2008). Clarithromycin vs ciprofloxacin as adjuncts to rifampicin and ethambutol in treating opportunist mycobacterial lung diseases and an assessment of Mycobacterium vaccae immunotherapy. Thorax.

[25] Park Y, Lee EH, Jung I, Park G, Kang YA (2019). Clinical characteristics and treatment outcomes of patients with macrolide-resistant Mycobacterium avium complex pulmonary disease: a systematic review and meta-analysis. Respir Res.

[26] Jarand J, Davis JP, Cowie RL, Field SK, Fisher DA (2016). Long-term follow-up of Mycobacterium avium complex lung disease in patients treated with regimens including clofazimine and/or rifampin. Chest.

[27] Wallace RJ, Brown-Elliott BA, McNulty S, Philley JV, Killingley J, Wilson RW, York DS, Shepherd S, Griffith DE (2014). Macrolide/azalide therapy for nodular/bronchiectatic Mycobacterium avium complex lung disease. Chest.

[28] Conyers LE, Saunders BM (2024). Treatment for non-tuberculous mycobacteria: challenges and prospects. Front Microbiol.

[29] Kim BG, Kim SR, Jhun BW (2024). Real-world outcomes of amikacin liposome inhalation suspension for refractory Mycobacterium avium complex pulmonary disease. Tuberc Respir Dis (Seoul).

[30] Haworth CS, Banks J, Capstick T, Fisher AJ, Gorsuch T, Laurenson IF, Leitch A, Loebinger MR, Milburn HJ, Nightingale M, Ormerod P, Shingadia D, Smith D, Whitehead N, Wilson R, Floto RA (2017). British Thoracic Society guidelines for the management of non-tuberculous mycobacterial pulmonary disease (NTM-PD). Thorax.

[31] Kim KJ, Oh SH, Jeon D, Chang CL (2023). Isolation and antimicrobial susceptibility of nontuberculous mycobacteria in a tertiary hospital in Korea, 2016 to 2020. Tuberc Respir Dis (Seoul).

[32] Luo J, Yu X, Jiang G, Fu Y, Huo F, Ma Y, Wang F, Shang Y, Liang Q, Xue Y, Huang H (2018). In vitro activity of clofazimine against nontuberculous mycobacteria isolated in Beijing, China. Antimicrob Agents Chemother.

[33] van Ingen J, Egelund EF, Levin A, Totten SE, Boeree MJ, Mouton JW, Aarnoutse RE, Heifets LB, Peloquin CA, Daley CL (2012). The pharmacokinetics and pharmacodynamics of pulmonary Mycobacterium avium complex disease treatment. Am J Respir Crit Care Med.

[34] Jeong BH, Jeon K, Park HY, Kim SY, Lee KS, Huh HJ, Ki CS, Lee NY, Shin SJ, Daley CL, Koh WJ (2015). Intermittent antibiotic therapy for nodular bronchiectatic Mycobacterium avium complex lung disease. Am J Respir Crit Care Med.

[35] Jung J, Chong YP, Lee HJ, Shim TS, Jo KW (2023). Comparison of treatment outcomes between intermittent and daily regimens in non-cavitary nodular bronchiectatic-type Mycobacterium avium complex pulmonary disease in relation to sputum smear results: a retrospective cohort study. Antimicrob Agents Chemother.

[36] Jeong BH, Jeon K, Park HY, Moon SM, Kim SY, Lee SY, Shin SJ, Daley CL, Koh WJ (2016). Peak plasma concentration of azithromycin and treatment responses in Mycobacterium avium complex lung disease. Antimicrob Agents Chemother.

[37] Loewenstein D, van Balveren L, Lemson A, Hanemaaijer N, Hoefsloot W, van Ingen J (2023). Monotherapy: key cause of macrolide-resistant Mycobacterium avium complex disease. Respir Med.

[38] Griffith DE, Brown-Elliott BA, Shepherd S, McLarty J, Griffith L, Wallace RJ (2005). Ethambutol ocular toxicity in treatment regimens for Mycobacterium avium complex lung disease. Am J Respir Crit Care Med.

[39] Schildkraut JA, Raaijmakers J, Aarnoutse R, Hoefsloot W, Wertheim HF, van Ingen J (2023). The role of rifampicin within the treatment of Mycobacterium avium pulmonary disease. Antimicrob Agents Chemother.

[40] van Ingen J, Hoefsloot W, Dartois V, Dick T (2024). Rifampicin has no role in treatment of Mycobacterium avium complex pulmonary disease and bactericidal sterilising drugs are needed: a viewpoint. Eur Respir J.

[41] Moon SM, Yoo IY, Huh HJ, Lee NY, Jhun BW (2019). Intermittent treatment with azithromycin and ethambutol for noncavitary mycobacterium avium complex pulmonary disease. Antimicrob Agents Chemother.

[42] Kim HJ, Lee JS, Kwak N, Cho J, Lee CH, Han SK, Yim JJ (2019). Role of ethambutol and rifampicin in the treatment of Mycobacterium avium complex pulmonary disease. BMC Pulm Med.

[43] Lee I, Hwang EJ, Kim JY, Yim JJ, Kwak N (2023). Treatment outcomes of clofazimine-containing regimens in severe Mycobacterium avium complex pulmonary disease. Open Forum Infect Dis.

[44] Zweijpfenning SM, Aarnoutse R, Boeree MJ, Magis-Escurra C, Stemkens R, Geurts B, van Ingen J, Hoefsloot W (2024). Safety and efficacy of clofazimine as an alternative for rifampicin in Mycobacterium avium complex pulmonary disease treatment: outcomes of a randomized trial. Chest.

[45] Stemkens R, Lemson A, Koele SE, Svensson EM, Te Brake LH, van Crevel R, Boeree MJ, Hoefsloot W, van Ingen J, Aarnoutse RE (2024). A loading dose of clofazimine to rapidly achieve steady-state-like concentrations in patients with nontuberculous mycobacterial disease. J Antimicrob Chemother.

[46] Kim SM, Chong YP, Lee HJ, Shim TS, Jo KW (2023). Comparison of treatment outcomes of cavitary Mycobacterium avium complex pulmonary disease with streptomycin or amikacin use. Microbiol Spectr.

[47] Kim BG, Kim H, Kwon OJ, Huh HJ, Lee NY, Baek SY, Sohn I, Jhun BW (2020). Outcomes of inhaled amikacin and clofazimine-containing regimens for treatment of refractory Mycobacterium avium complex pulmonary disease. J Clin Med.

[48] Jhun BW, Kim SY, Moon SM, Jeon K, Kwon OJ, Huh HJ, Ki CS, Lee NY, Shin SJ, Daley CL, Koh WJ (2018). Development of macrolide resistance and reinfection in refractory Mycobacterium avium complex lung disease. Am J Respir Crit Care Med.

[49] Jhun BW, Moon SM, Kim SY, Park HY, Jeon K, Kwon OJ, Huh HJ, Ki CS, Lee NY, Chung MJ, Lee KS, Shin SJ, Daley CL, Koh WJ (2018). Intermittent antibiotic therapy for recurrent nodular bronchiectatic Mycobacterium avium complex lung disease. Antimicrob Agents Chemother.

[50] Kim HJ (2024). Nonpharmacological treatment for nontuberculous mycobacterial pulmonary disease. Tuberc Respir Dis (Seoul).

[51] Griffith DE, Eagle G, Thomson R, Aksamit TR, Hasegawa N, Morimoto K, Addrizzo-Harris DJ, O'Donnell AE, Marras TK, Flume PA, Loebinger MR, Morgan L, Codecasa LR, Hill AT, Ruoss SJ, Yim JJ, Ringshausen FC, Field SK, Philley JV, Wallace RJ, van Ingen J, Coulter C, Nezamis J, Winthrop KL; CONVERT Study Group (2018). Amikacin liposome inhalation suspension for treatment-refractory lung disease caused by Mycobacterium avium complex (CONVERT): a prospective, open-label, randomized study. Am J Respir Crit Care Med.

[52] Philley JV, Wallace RJ, Benwill JL, Taskar V, Brown-Elliott BA, Thakkar F, Aksamit TR, Griffith DE (2015). Preliminary results of bedaquiline as salvage therapy for patients with nontuberculous mycobacterial lung disease. Chest.

[53] Mitchell JD (2019). Surgical treatment of pulmonary nontuberculous mycobacterial infections. Thorac Surg Clin.

[54] Kim JY, Lee HW, Yim JJ, Kwak N (2023). Outcomes of adjunctive surgery in patients with nontuberculous mycobacterial pulmonary disease: a systematic review and meta-analysis. Chest.

[55] Kim Y, Yoon JH, Ryu J, Yang B, Chung SJ, Kang HK, Park DW, Park TS, Moon JY, Kim TH, Kim SH, Sohn JW, Yoon HJ, Lee H, Choi H (2023). Gastroesophageal reflux disease increases susceptibility to nontuberculous mycobacterial pulmonary disease. Chest.

[56] Kwak N, Choi H, Jeon D, Jhun BW, Jo KW, Kang YA, Kwon YS, Lee M, Mok J, Shim TS, Shin HJ, Whang J, Yim JJ (2020). Protocol of a nationwide observational study among patients with nontuberculous mycobacterium pulmonary disease in South Korea (NTM-KOREA). Tuberc Respir Dis (Seoul).

